# Inhibitory Effect of (2*R*)-4-(4-hydroxyphenyl)-2-butanol 2-*O*-β-d-apiofuranosyl-(1→6)-β-d-glucopyranoside on RANKL-Induced Osteoclast Differentiation and ROS Generation in Macrophages

**DOI:** 10.3390/ijms22010222

**Published:** 2020-12-28

**Authors:** Eun-Nam Kim, Ga-Ram Kim, Jae Sik Yu, Ki Hyun Kim, Gil-Saeng Jeong

**Affiliations:** 1 College of Pharmacy, Keimyung University, Daegu 42601, Korea; enkimpharm@gmail.com (E.-N.K.); o930302@naver.com (G.-R.K.); 2 School of Pharmacy, Sungkyunkwan University, Suwon 16419, Korea; jsyu@bu.edu

**Keywords:** *Betula platyphylla* var. *japonica*, reactive oxygen species (ROS), superoxide dismutase (SOD), catalase (CAT), osteoclast

## Abstract

In bone homeostasis, bone loss due to excessive osteoclasts and inflammation or osteolysis in the bone formation process cause bone diseases such as osteoporosis. Suppressing the accompanying oxidative stress such as ROS in this process is an important treatment strategy for bone disease. Therefore, in this study, the effect of (2*R*)-4-(4-hydroxyphenyl)-2-butanol 2-*O*-β-d-apiofuranosyl-(1→6)-β-d-glucopyranoside (BAG), an arylbutanoid glycoside isolated from *Betula platyphylla* var. *japonica* was investigated in RANKL-induced RAW264.7 cells and LPS-stimulated MC3E3-T1 cells. BAG inhibited the activity of TRAP, an important marker of osteoclast differentiation and F-actin ring formation, which has osteospecific structure. In addition, the protein and gene levels were suppressed of integrin β3 and CCL4, which play an important role in the osteoclast-induced bone resorption and migration of osteoclasts, and inhibited the production of ROS and restored the expression of antioxidant enzymes such as SOD and CAT lost by RANKL. The inhibitory effect of BAG on osteoclast differentiation and ROS production appears to be due to the inhibition of MAPKs phosphorylation and NF-κβ translocation, which play a major role in osteoclast differentiation. In addition, BAG inhibited ROS generated by LPS and effectively restores the mineralization of lost osteoblasts, thereby showing the effect of bone formation in the inflammatory situation accompanying bone loss by excessive osteoclasts, suggesting its potential as a new natural product-derived bone disease treatment.

## 1. Introduction

The balance between osteoclasts and osteoblasts mediating bone resorption and formation plays a very important role in bone health [1,2]. Bone is constantly destroyed and replaced to maintain homeostasis, and this imbalance of homeostasis is one of the causes of osteoporosis. Osteoporosis is characterized by an increased risk of fracture due to microarchitectural damage resulting from a decrease in bone mass; the patient does not know, because it occurs silently and gradually, without symptoms or signs, until a fracture occurs [3,4]. It is a major public health problem throughout the world as the incidence of osteoporosis increases yearly with age [3,5]. Osteoporosis is mainly caused by excessive bone resorption due to excessive osteoclastogenesis. Therefore, most osteoporosis therapy has been focused on the development of drugs that block bone resorption by reducing the formation or activity of osteoclasts. Bisphosphonate not only has a direct apoptosis effect on osteoclasts but also acts as a powerful inhibitor of bone resorption by inhibiting osteoclastogenesis and maturation [6]. Bisphosphonates have a higher binding affinity to the bone than other antiresorptive agents, so they can persist in the bone and remainsexposed to the pharmacological effects of the drug for several years after the patient stops the drug [7]. However, this drug causes serious side effects, such as jaw bone necrosis and atypical femur fractures [7]. Therefore, it is important to develop natural therapeutics that can replace these side effects.

Reactive oxygen species (ROS), known as cytotoxicity and damage, are known to play an important role in regulating the molecular signals of osteoclast differentiation, and ROS regulates osteoclast differentiation by regulating the RANK signaling pathway [8,9,10]. During osteoclast differentiation, RANKL can increase the level of intracellular ROS by activating signaling processes including tumor necrosis factor receptor (TNFR)-associated factor 6 (TRAF6) and NADPH oxidase 1 (Nox1) [10]. Meanwhile, according to previous reports, oxidative stress was generated to promote activation of the MAPK (ERK, JNK, p38) pathway, a downstream signal by RANKL [11]. The production of ROS by RANKL-induced osteoclasts is known to inhibit the expression of antioxidant proteins such as catalase (CAT) and superoxide dismutase (SOD) [12]. Therefore, ROS can be considered an important signaling messenger in the osteogenesis process induced by RANKL, but its role has not been studied yet.

*Betula platyphylla* var. *japonica* (Miq.) H. Hara, a deciduous broad-leaved tree, is widely distributed not only in East Asia but also in Sakhalin and Siberia [13,14]. In China’s Sinnongbon Carbide and Korea’s Donguibogam, the bark of *B. platyphylla* var. *japonica* has been used for folk medicine under the name Baekhwapi (白樺皮) or Hwapi (樺皮) [15]. Among the reported compounds present in the shell of *B. platyphylla* var. *japonica*, betulin has been reported to inhibit cancer cells or osteoclast differentiation [16]. Betulin has also been isolated from the barks of *B. platyphylla* var. *japonica*, and it has been reported to suppress osteoclastogenesis [17]. In the current study, chemical investigation of barks of *B. platyphylla* var. *japonica* led to the isolation of an arylbutanoid glycoside, (2*R*)-4-(4-hydroxyphenyl)-2-butanol 2-O-β-d-apiofuranosyl-(1→6)-β-d-glucopyranoside (BAG). In previous pharmacological studies, BAG showed significantly inhibitory activity on lipid peroxidation as well as α-tocopherol, BHA and BHT [18] and also exhibited melanogenesis-inhibitory activity with no toxicity to the B16 melanoma cells [19]. BAG was reported to show remarkable inhibitory activity against the degranulation of RBL-2H3 by antigen stimulation, which can potentially be used for the treatment of IgE–FcεRI interaction-related allergic disorders [20]. Recently, BAG was found to show inhibition of adipocyte differentiation and regulation of metabolic genes in mature adipocytes, which can be beneficial effects in the regulation of adipocyte differentiation and adipocyte metabolism [21], however, its effects on osteoclast differentiation or bone formation have rarely been studied. As a therapeutic agent developed to date, inhibitors that suppress excessive bone resorption of osteoclasts are used, but when osteoclasts function worse, osteoblasts also have a decreased osteogenic function, so the therapeutic effect is not significant. Therefore, in this study, we explored the effects of BAG isolated from *B. platyphylla* var. *japonica* on osteoclasts and osteoblasts and the effects of BAG on ROS in the process.

## 2. Results

### 2.1. Effects of BAG on Cytotoxicity and Cell Confluency in RAW264.7 Cells

**Figure 1:**
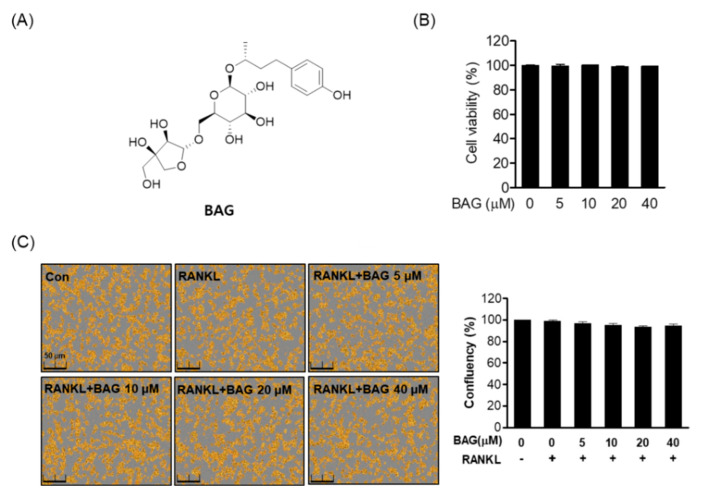
Effects of BAG on cytotoxicity and cell confluency in RAW264.7 cells. (**A**) The chemical structure and formula of BAG. (**B**) RAW264.7 cells (5 × 10^3^ cell/mL) were seeded for 24 h and then treated with BAG at the indicated concentration (5–40 μM) in 96-well plates for 5 days, and viability was measured by MTT assay. (**C**) The confluency (%) of RAW 264.7 cells was determined using the Incucyte^®^ Live-Cell analysis imaging system.

Before the experiment, we first determined whether BAG (Figure 1A) was cytotoxic to RAW264.7. As a result of MTT analysis, BAG did not show cytotoxicity for 5 days between the indicated concentrations (5–40 μM) (Figure 1B). In addition, it was confirmed through Incucyte^®^ Live-Cell analysis systems that RAW264.7 cells treated with RANKL at the indicated concentration did not affect cell confluency (Figure 1C).

### 2.2. Inhibitory Effect of BAG on RANKL Induced Osteoclast Differentiation

**Figure 2:**
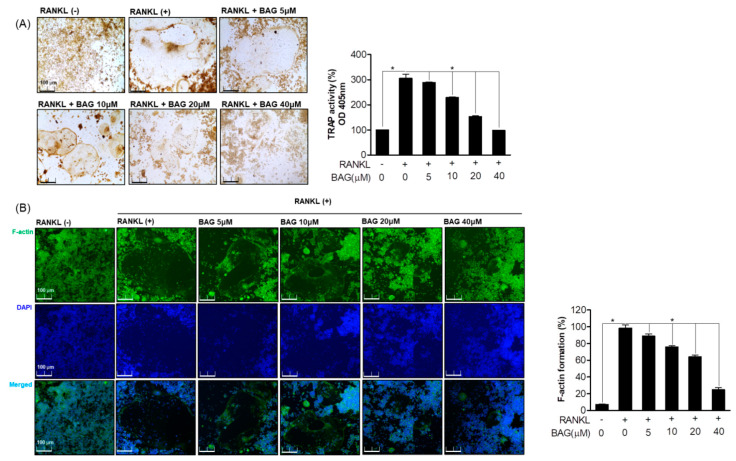
Inhibitory Effect of BAG on RANKL induced osteoclast differentiation. (**A**) RAW264.7 cells were seeded in a 24 well plate at a density of 5 x 10^3^ and treated with RANKL with various concentrations of BAG (5–40 μM) for 5 days, and then TRAP staining and activity were measured. (**B**) As a representative fluorescence micrograph of the effect of BAG on F-actin belt formation, the indicated concentrations of BAG (5–40 μM) were treated with RANKL for 5 days. The actin cytoskeleton was then stained with Alexa 488-Phalloidin (green), and the nuclei were counterstained with DAPI (blue). * *p* < 0.05, versus the RANKL treated group.

To evaluate the effect of BAG on the induction of osteoclast formation by RANKL, a negative control and RANKL treatment or BAG treatment with RANKL were done. To evaluate the effect of BAG on the induction of osteoclast formation by RANKL, the TRAP staining and activity of the negative control group and the RANKL-treated group or the group treated with RANKL and BAG were evaluated. As a result, the generation and activity of TRAP by RANKL were promoted, and the production and activity of TRAP were inhibited in a concentration-dependent manner by treatment with BAG (Figure 2A). To evaluate the effect of BAG on the production and activity of TRAP, as well as to evaluate the morphological changes to the F-actin skeletal structure, a structural feature of mature osteoclasts, it was first stained with Alexa 488-phalloidin. It was confirmed that the area of the inactive podosomal actin belt surrounding the cells of mature osteoclasts induced by RANKL was reduced by BAG in a concentration-dependent manner (Figure 2B). These results suggest that BAG has an inhibitory effect on the differentiation process of osteoclasts and maturation of osteoclasts by RANKL.

### 2.3. Inhibitory Effect of BAG on RANKL Induced Osteoclast of Functions

**Figure 3:**
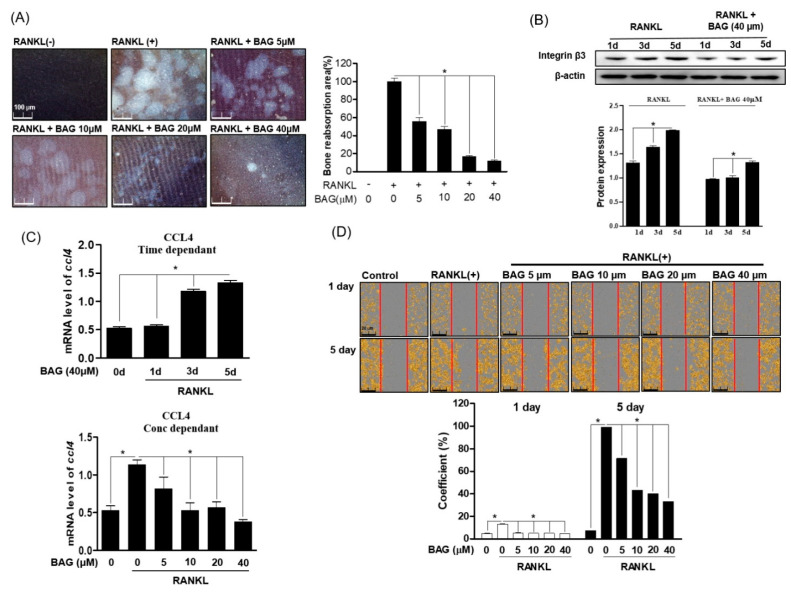
Inhibitory Effect of BAG on RANKL induced osteoclast of functions. (**A**) RAW264.7 cells (5 × 10^3^) were seeded, then RANKL (50 ng/mL) with or without the indicated concentration of BAG (5–40 mM) was placed in the osteo-surface well for 5 days. The percentage of bone resorption pit area relative to the total osteo-surface well area was quantified for the experimental condition. RAW 264.7 cells were cultured in the RANKL with or without BAG for 1, 3, and 5 days to induce osteoclast differentiation, and the protein expression of integrin β3 was measured by Western blot (**B**), and the levels of CCL4 mRNA were measured by real-time PCR (**C**). The number of migrated cells with confluency average using Incucyte^®^ Live-Cell analysis imaging system (**D**). * *p* < 0.05, versus the RANKL-treated group.

We evaluated the effect of BAG on osteoclast differentiation as well as bone resorption and migration, which are the main functions of osteoclasts. The ability to resorb and invade bones is the core behavior of osteoclasts, and CCL4 and integrin β3 play an important role in regulating the invasive behavior of osteoclasts. First, in order to evaluate the effect of BAG on the bone resorption of RANKL-induced osteoclasts, RAW264.7 cells were cultured in an osteo-surface well and treated with RANKL at the indicated concentration to induce osteoclasts, and at the same time BAG (5–40 μM) was treated for 7 days. After removing the cells, as a result of evaluating the bone resorption area of the osteo-surface well by differentiated osteoclasts, it was confirmed that the area of bone loss by the osteoclasts differentiated by RANKL decreased with BAG treatment (Figure 3A). In addition, as a result of evaluating the levels of Integrin β3 protein expression (Figure 3B) and CCL4 (Figure 3C) gene in the RANKL-only treated group and BAG with RANKL together treated group for 1, 3 and 5 days, the expression of proteins and genes involved in this osteoclast migration by BAG were evaluated, and it was confirmed that the level was suppressed. To evaluate the effect of BAG on the migration of differentiated osteoclasts by RANKL treatment, migration analysis was performed by introducing scratches into the confluence layer of cells. In order to evaluate the effect of BAG on the migration of differentiated osteoclasts by RANKL treatment, migration analysis was performed by introducing a scratch to the confluence layer of cells, and BAG reduced the actual migration area of osteoclasts in a concentration-dependent manner (Figure 3D). These results suggest the potential of BAG as a regulator to inhibit the differentiation, formation and function of osteoclasts.

### 2.4. BAG Regulates the Generation of RANKL-Induced ROS and the Expression of Antioxidant Genes

**Figure 4:**
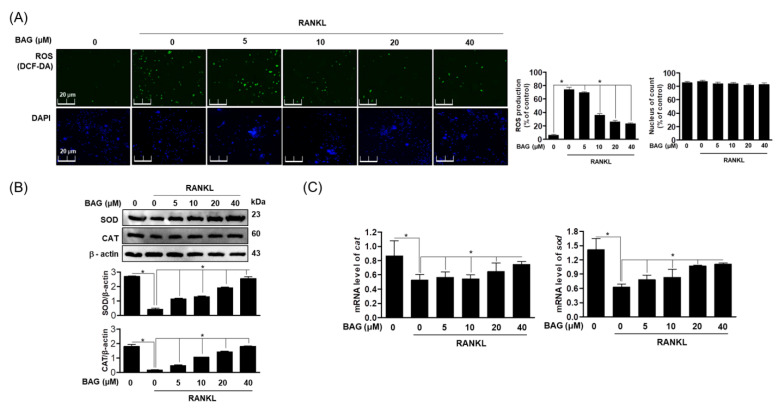
BAG regulates the generation of RANKL-induced ROS and the expression of antioxidant genes. (**A**) Representative stain images of RANKL-induced ROS generation detected by DCFH-DA (green) and immunofluorescence for nuclei using DAPI (blue) in RAW264.7 with different indicated concentrations of BAG. (**B**) The expressions of SOD and CAT antioxidant proteins were analyzed by Western blot analysis from cells pre-treated with BAG and stimulated by RANKL (50 ng/mL). (**C**) The mRNA level of antioxidant genes was measured by real-time PCR. * *p* < 0.05, versus the RANKL treated group.

Previously research has shown that RANKL-induced ROS plays a role as an important secondary messenger in regulating osteoclast differentiation [22]. Therefore, in order to understand the basic molecular mechanism of BAG in RANKL-induced ROS production, we used DCF-DA to evaluate the accumulation of reactive oxygen species (ROS) in RANKL-stimulated cells and the resulting effect on proteins and genes of antioxidant enzymes. First, it was confirmed that the accumulation of ROS increased with osteoclast differentiation by RANKL, and it was confirmed that the level of ROS was suppressed in a concentration-dependent manner by BAG treatment (Figure 4A). In addition, BAG recovered protein expression (Figure 4B) and gene level (Figure 4C) of the antioxidant enzymes Superoxide dismutase (SOD) and catalase (CAT), which were lost due to ROS production by RANKL, in a concentration-dependent manner. These results suggest that BAG can be used as an antioxidant modulator and inhibit osteoclast differentiation by regulating antioxidant enzymes through ROS regulation in the process of osteoclast differentiation by RANKL.

### 2.5. BAG Downregulates RANKL-Induced Expression of Osteospecific Protein and Gene

**Figure 5:**
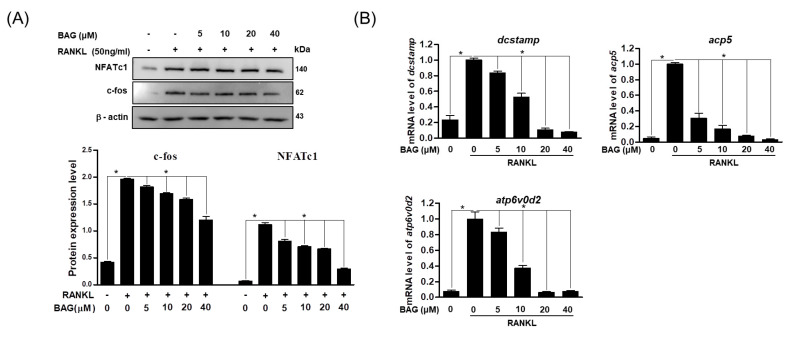
BAG downregulates RANKL-induced expression of osteoclast specific protein and gene. (**A**) For total proteins from RAW264.7 stimulated with RANKL without or with indicated concentration for 24 h, Western blot analyses were performed using specific antibodies against c-Fos and NFATc1. The protein expression of c-Fos and NFATc1 relative to b-actin were quantified by densitometry. (**B**) The mRNA level of osteoclast-specific genes was measured by real-time PCR. * *p* < 0.05 versus the RANKL-treated group.

NFATc1 and c-Fos are known as regulators of osteoclast formation [23]. Therefore, in order to confirm the mechanism for the inhibitory effect of BAG on osteoclast differentiation, the expression levels of NFATc1 and c-Fos, which are known as regulatory markers of osteoclasts, and corresponding osteoclast-specific genes were investigated. After treatment of RAW264.7 cells with RANKL or RANKL + BAG (indicated concentration), the changes of these proteins were confirmed by Western blot assay. As a result, it was confirmed that proteins of NFATc1 and c-Fos increased by RANKL and decreased in a concentration-dependent manner by BAG treatment (Figure 5A). In addition, the effect of inhibition of RANKL-induced NFATc1 and c-Fos activation by BAG on osteospecific genes such as dendritic cell-specific transmembrane protein (DC-STAMP), acid phosphatase 5 (ACP5) and ATP6v0d2 was analyzed by real time-PCR. As a result, it was found that the level of osteoclast-specific genes was also reduced by the concentration-dependent treatment of BAG (Figure 5B). Therefore, in the process of osteoclast differentiation by RANKL, BAG is thought to exhibit an inhibitory effect on osteoclast differentiation by inhibiting the signal transduction essential for osteoclast differentiation and the expression of downstream proteins and genes in the early stages of differentiation.

### 2.6. BAG Suppresses RANKL-Induced Activation of NF-kβ and MAPKs

**Figure 6:**
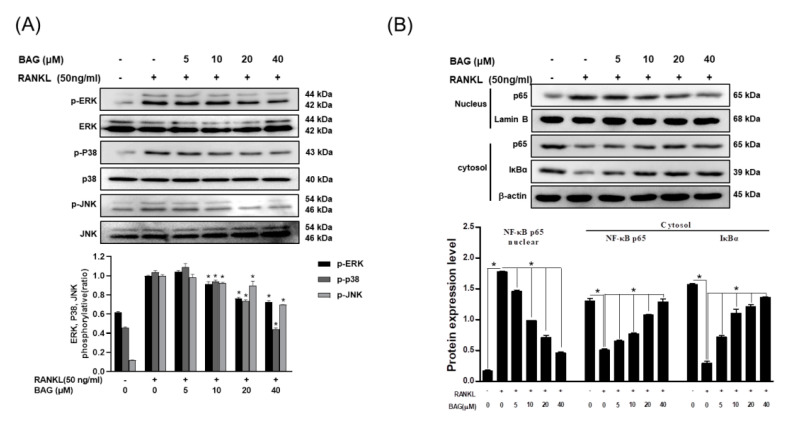
BAG suppresses RANKL-induced activation of NF-kβ and MAPKs. (**A**) RAW264.7 cells (5 × 10^5^ cell/mL) were seeded for 24 h, then pre-incubated with the indicated concentration of BAG for 1 h and stimulated with 50 ng/mL of RANKL for 30 min and were subjected to Western blot analyses using specific antibodies’ phosphorylated forms of ERK, p38, and JNK. (**B**) NE-PER (Nuclear and Cytoplasmic Extraction Reagents) kits were used for separation of cytoplasm and nucleus, and Western blot analysis was performed on each cytoplasm and nucleus. Graphs show normalized expression of each indicated protein against the expression of β-actin or lamin B. * *p* < 0.05 versus the RANKL-treated group.

In the previous results, BAG inhibited the expression of NFATc1 and c-Fos upregulated by RANKL in a concentration-dependent manner. Therefore, the effect of BAG on mitogen-activated protein kinase (MAPK) and nuclear factor kappa-light-chain-enhancer of activated B (NF-κβ), the upper signals of these proteins, was evaluated through Western blot assay. First, RANKL-induced RAW264.7 cells increased the phosphorylation activity of MAPKs (ERK, p38, JNK), and the phosphorylation activity of ERK, p38 and JNK was inhibited in a concentration-dependent manner by treatment with BAG (Figure 6A). In addition, the translocation of p65, a subunit of NF-κβ, has already been reported in several studies and described as a basic mechanism for osteoclast differentiation. Therefore, the role of BAG in the nuclear translocation of NF-κβ was evaluated by Western blot assay. As a result, BAG inhibited the expression of NF-κβ p65 increased by RANKL in the nucleus in a concentration-dependent manner, which led to an increase in the expression of p65 and IκBα in the cytosol (Figure 6B). These results suggest that BAG exhibits an inhibitory effect on osteoclast differentiation through the inhibition of MAPKs phosphorylation and nuclear translocation of NF-κβ, which are the basic mechanisms for osteoclast differentiation.

### 2.7. BAG Induces Bone Formation through inhiBition of LPS-Induced ROS and Up-Expression of Osteoblast-Specific Genes

**Figure 7:**
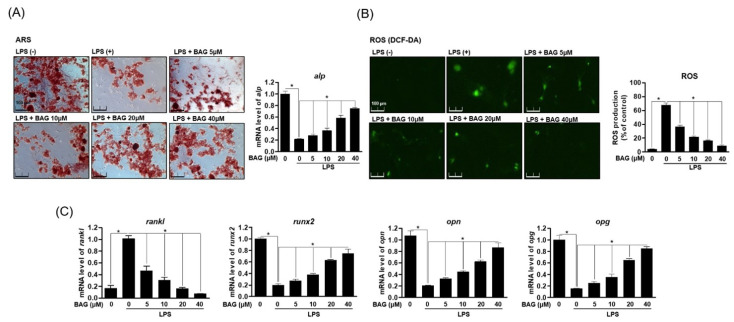
BAG induces bone formation through inhibition of LPS-induced ROS and up-expression of osteoblast-specific genes. (**A**) MC3T3-E1 cells were cultured for 14 days with a medium containing 50 μg/mL ascorbic acid and 20 mM β-glycerophosphate and simultaneously treated with the indicated concentrations of BAG and LPS, followed by Alizarin red s staining. (**B**) Representative stain images of LPS-induced ROS generation were detected by using DCFH-DA in MC3T3-E1 cells with different indicated concentrations of BAG. (**C**) Osteoblast-specific gene expression was analyzed using real-time PCR, and the results were normalized to the expression of GAPDH. * *p* < 0.05, versus the RANKL treated group.

The balance of bone absorption and formation is a very important physiological condition for bone health. In the above results, BAG regulated the expression of antioxidant enzymes by down-regulating ROS induced by RANKL and effectively inhibited the differentiation and function of osteoclasts. Therefore, we explored the role of BAG in LPS-stimulated osteoblasts. First, MC3T3-E1 cells were differentiated into osteoblast differentiation medium and treated with LPS without or with BAG. After that, ALP (alkaline phosphatase) was measured through alizarin red s staining, an important index of osteoblast differentiation. As a result, it was found that the differentiation ability of osteoblasts lost by LPS treatment was restored by treatment with BAG (Figure 7A). In addition, the effect was confirmed of BAG on osteoblast-specific genes on the generation of ROS by LPS in MC3T3-E1 cells. As a result, in the LPS-induced MC3T3-E1 cells, BAG down-regulated the production of ROS in a concentration-dependent manner (Figure 7B), inhibited the increased RANKL gene and at the same time promoted the recovery of the lost osteoblast-specific genes (RUNX2, OPN, OPG) (Figure 7B). It is believed that BAG affects osteoclast differentiation and ROS production, as well as osteoblast differentiation and proliferation.

## 3. Discussion

Bone is a dynamic tissue maintained by osteoclasts that bone resorption and osteoblasts that create new bones. Osteoclasts that are abnormally formed excessively destroy the microstructure of the bone, causing diseases such as osteoporosis and the beginning is regulated by receptor activator of nuclear factor kβ (NF-kβ) ligand (RANKL)/RANK binding signals [24,25]. Therefore, this study evaluated the effect of compound BAG isolated from *B. platyphylla* on osteoclasts and osteoblasts. Bone loss and resorption by oxygen free radicals during RANKL-induced osteoclast differentiation have been previously reported in studies, and excessive generation of ROS can induce oxidative stress and cause various diseases such as neurodegenerative diseases and aging [26,27]. ROS generated by RANKL induction can play a role in osteoclast formation as a secondary messenger during osteoclastogenesis, and osteoclasts by RANKL express osteoclast-related genes such as TRAP, ACP5, DC-STAMP and ATP6v0d2 [28]. In the results of this study, BAG effectively suppressed the expression of these osteoclast-specific genes (TRAP, DC-STAMP and ATP6v0d2) and their important functions, bone resorption and the expression of important proteins and genes involved in the migration and proliferation of osteoclasts such as Integrin β3 and chemokine CC motif ligand 4 (CCL4).

In a previous study, phosphorylation of MAPKs and translocation of NF-kβ during RANKL induced osteoclast differentiation and decisively induced the formation of TRAP-positive osteoclasts through c-Fos and NFATc1 expression [29,30]. In addition, during osteoclastogenesis, RANKL binds to the receptor RANK and produces intracellular ROS through NADPH oxidase 1 (NOX1) production through the activation of tumor necrosis factor (TNF) receptor-associated factor 6 (TRAF6) [10]. Therefore, in this study, the effect of BAG on ROS production in cells was evaluated in the process of inhibiting the differentiation and function of RANKL-induced osteoclasts. As a result, BAG inhibited osteoclast differentiation and also inhibited ROS production, thereby recovering protein and gene levels of antioxidant enzymes such as SOD and CAT, which were lost by RANKL. In addition, BAG inhibited the phosphorylation of MAPKs and translocation of NF-κβ, and these results inhibited the expression of c-Fos and NFATc1, which are downstream signals that play a crucial role in the osteoclast differentiation process. These results suggest that BAG effectively regulates proteins and genes involved in the process of osteoclast differentiation induced by RANKL, thereby inhibiting osteoclast differentiation and its function. At the same time, by regulating ROS and the inflammatory situation accompanied by osteoclast differentiation, it is also expected to be effective as an anti-inflammatory modulator.

In this study, since not only the role of osteoclasts but also of osteoblasts is important in bone homeostasis, the effects of BAG on osteoblasts as well as osteoclasts were studied. Previously reported studies have shown that Gram-negative bacterial lipopolysaccharide (LPS) has an important correlation with bone loss due to excessive osteoclasts and destructive damage to bone tissue in osteoporosis or bone disease [31] and reported a significant decrease in osteogenic differentiation even in MC3T3-E1 exposed to LPS [32]. It is known that inhibition of osteoblast differentiation by LPS significantly reduces the expression of osteoblast-differentiation-specific genes such as RUNX2, OPN and OPG [33]. The results of this study showed that BAG restored the mineralization of osteoblasts lost by LPS during the differentiation process of osteoblasts and restored osteoblast differentiation by lowering the level of ROS in inflammatory stimulation such as LPS. In addition, BAG suppressed the gene level of RANKL increased by LPS and showed a result of recovering the level of the lost osteoblast-specific gene.

Therefore, to summarize the results of this study, BAG isolated from *B. plantyphylla* effectively regulated bone resorption due to excessive osteoclasts and oxidative stress such as ROS in RANKL-stimulated RAW264.7, and loss of osteoblasts caused by LPS. In the process, it showed the effect of promoting osteoblastogenesis along with the recovery of specific genes of osteoblasts. In conclusion, this research suggests the possibility of BAG as a new natural treatment for osteoporosis or osteolytic disease.

## 4. Materials and Methods

### 4.1. Reagents and Chemicals

Recombinant soluble RANKL ligand (sRANKL) was acquired from PeproTech EC Ltd. (London, UK). Acid Phosphatase Assay kit, 3-(4,5-Dimethylthiazol-2-yl)-2,5-diphenyltetrazoliumbromide (MTT), Leukocyte acid phosphatase and 4-6-Diamidino-2-phenylindole (DAPI), ascorbic acid, β-glycerophosphate, Lipopolysaccharides (LPS) and Alizarin Red S was purchased from Sigma-Aldrich Fine Chemicals (Saint Louis, MO, USA). Dulbecco’s modified Eagle’s medium (DMEM), Minimum essential medium alpha (α-MEM) and fetal bovine serum (FBS) was purchased from Welgene Bioscience (Daegu, Korea). TRIZOL reagent was purchased from JBI (Seoul, Korea), and TOPscript™ RT DryMIX (dT18 plus) was purchased from Enzynomics (Daejeon, Korea). TB Green^®^ Premix Ex Taq™ II (Tli RNaseH Plus) was purchased from Takara (Tokyo, Japan). Primary antibodies for phospho-JNK, JNK, phospho-p38, p38, phospho-IĸBα, IĸBα and rabbit polyclonal antibodies were purchased from Cell Signaling Technology Inc. (Danvers, MA, USA). Phospho-ERK, ERK, p65 and anti-c-Fos antibodies were obtained from Santa Cruz Biotechnology (Dallas, TX, USA). An anti-nuclear factor of activated T cells-c1 (NFATc1), catalase (CAT) and superoxide dismutase (SOD) antibody were acquired from BD biosciences (San Jose, CA, USA). The enhanced chemiluminescence (ECL) Western blotting detection system was purchased from Advansta Inc. (San Jose, CA, USA). The Osteo Assay Plate was purchased from Corning Inc. (Corning, NY, USA). Acid Phosphatase 5, Tartrate Resistant (ACP5), ATPase H+ Transporting V0 Subunit D2 (ATP6V0d2) and dendritic cell specific transmembrane protein (DC-STAMP), osteopontin (OPN), alkaline phosphatase (ALP), runt-related transcription factor 2 (RUNX2), Osteoprotegerin (OPG), catalase (CAT), superoxide dismutase (SOD) and receptor activator of nuclear factor kappa-B ligand (RANKL) primer was purchased from Biomedic Co., (Bucheon, Korea).

### 4.2. Isolation and Identification of BAG from the Barks of B. platyphylla var. japonica

The dried barks of *B. platyphylla* var. *japonica* (200.0 g) were extracted with 80% EtOH at room temperature for 24 h. The extracts were filtered and concentrated under reduced vacuo to provide a crude extract (17.0 g). Thereafter, the extract was suspended in hot H_2_O, and the resulting H_2_O layer was partitioned with CHCI_3_, EtOAc, *n*-buOH to obtain fractions of 12.5 g, 1.3 g and 1.5 g, respectively. The *n*-BuOH soluble fraction (1.5 g) was subjected to silica gel column chromatography eluting with CH_2_CI_2_:MeOH:H_2_O (20:4.5:0.5, 30:10:1 and 10:5:1) solvent conditions to obtain ten fractions (A1–A10). Subsequently, A7 (380 mg) was subjected to silica gel column chromatography eluting with MeOH:H_2_O (4:6 and 1:0) solvent conditions to give five fractions (A71–A75). A73 (130 mg) was subjected to semi-prepative high-pressure liquid chromatography (HPLC) eluting with 40% MeOH solvent condition to isolate compound 1 (30 mg). The purity of compound 1 (>95.2%) was evaluated by HPLC analysis. For structural identification of compound 1 nuclear magnetic resonance (NMR) data was measured on Bruker AVANCE III 700 NMR of Bruker (Karlsruhe, Germany). NMR solvent CD_3_OD was purchased from Sigma-Aldrich (Saint Louis, MO, USA). Compound 1 was determined as BAG (Figure 1A) by comparing data from previously reported NMR analysis data (Table 1) literature [34].

### 4.3. Cell Culture and Differentiation

RAW 264.7 murine macrophage cells were acquired from the American Type Culture Collection (ATCC, Rockville, MD, USA), the RAW 264.7 cells were cultured in Dulbecco’s modified Eagle’s medium (DMEM) containing 10% fetal bovine serum (FBS), 100 U/mL of penicillin and streptomycin. MC3T3-E1 mouse cell line was also purchased from the ATCC, and the MC3T3-E1 cells were cultured in Minimum essential medium alpha (α-MEM) containing 10% FBS, 100 U/mL of penicillin and streptomycin. Cells were incubated in a humidified atmosphere with 5% CO_2_.

RAW 264.7 cells were seeded at 5 × 10^3^ cells/well in 24-well plates and incubated 24 h. Then, α-MEM with 10% FBS and 100 U/mL of penicillin and streptomycin was refreshed to each well. After being treated or untreated with RANKL (50 ng/mL), they were cultured for 5 days. The medium was refreshed every day. After 5 days, osteoclasts were identified by TRAP or several experimental methods. MC3T3-E1 mouse cells were seeded at 3 × 10^3^ cells/well in 24-well plates and incubated 24 h. Then, α-MEM with 50 mg/mL ascorbic acid and 50 mM β-glycerophosphate was refreshed for 14 days.

### 4.4. MTT Assay

Cell viability was measured according to the following method. RAW 264.7 cells were seeded at 5 × 10^3^ cells/well in 96-well plates. After samples were processed or unprocessed, cells were cultured for 24 h. Then, 4,5-dimethylthiazol-2-thiazolyl)-5-diphenyltetrazolium bromide (MTT) (5 mg/mL) in each well was added and incubated for 4 h. After 4 h, MTT solvent was removed, the cells were lysed in Dimethyl Sulfoxide (DMSO) and the cell viability was decided by measuring absorbance at 540 nm in a microplate reader (TECAN infinity pro 2000, Männedorf, Switzerland). Cell viability was expressed as a percentage of unprocessed control cells. Each group was analyzed separately 3 times.

### 4.5. TRAP Activity and Staining

In this experiment, Acid Phosphatase Assay kit was used to confirm TRAP activity. After picking and dispensing the supernatant in 96 wells, the substrate was dispensed and reacted for 20 min in an incubator. Then, a stop solution was added to stop the reaction, and the absorbance was measured at 405 nm. RAW 264.7 cells were dispensed in 24 wells in DMEM medium containing FBS and penicillin, and after 24 h, the medium was replaced with a α-MEM medium containing 50 ng/mL RANKL. Differentiation medium was changed every 2 days. After 7 days, the medium was removed and washed twice with phosphate-buffered saline (PBS). Cells were fixed with 4% formaldehyde for 15 min and washed with PBS. Leukocyte acid phosphatase kit was used to react with fixed cells at 37 °C with 5% CO_2_ for 1 h and then reacted in a shaded state. Thereafter, the cells were washed three times with PBS, and TRAP-positive multinuclear cells including three or more nuclei were measured with an optical microscope.

### 4.6. Actin Ring and DAPI Staining

The degree of differentiation of osteoclasts was measured by staining the ring part with Allexa 488. Specifically, RAW 264.7 cells were incubated with processed or unprocessed compounds for 5 days. The cells were fixed in 4% formaldehyde for 15 min, before treatment with 0.5% Triton X-100. Subsequently, the cells were stained with Allexa 488 for 1 h, followed by contrasted staining with DAPI for 30 min. This process was conducted in the dark. After staining, they were washed using cold PBS. Actin rings and DAPI staining in mature osteoclasts were visualized with a fluorescence microscope (Nikon Co., Tokyo, Japan).

### 4.7. Pit Formation Assay

RAW 264.7 cells were dispensed at a density of 1 × 10^3^ cells/well on a pit formation assay plate (Corning, NY, USA) and cultured for 24 h. After 24 h, they were refreshed with α-MEM medium containing 5% FBS and treated or untreated with RANKL (50 ng/mL) and Sample to incubate for 14 days. The medium was refreshed every day. The medium was then removed and washed twice with PBS. Cells were removed by treatment with 5% sodium hypochlorite for 5 min, washed with PBS and dried. Finally, the area of each well was photographed using a microscope and measured with an Image J program.

### 4.8. Measurement of ROS Production

RAW264.7 was incubated in a 24-well plate at a concentration of 5 × 10^3^ cells/well for 24 h. Then, after RANKL treatment, pretreatment was performed with the indicated concentration of BAG for 24 h. MC3T3-E1 was incubated in a 24-well plate at a concentration of 1 × 10^3^ cells/well for 24 h. Then, the indicated concentrations of BAG and LPS were treated for 14 days with the differentiation medium specified in 4.3. After that, in order to evaluate the production of ROS, 2′, 7′-dichlorodihydrofluorescein diacetate (DCF-DA) was used and incubated for 20 min at 37 °C in the dark. After 20 min, cells were washed with PBS and fixed with 4% paraformaldehyde (pH 7.4) for 20 min. After observation, ROS was detected with a fluorescent Olympus IX microscope 71-F3 2PH (Tokyo, Japan).

### 4.9. Cell Migration

RAW264.7 cells (1 × 10^3^ cells/well) were incubated in a 24-well plate for 24 h, followed by constant scratching. Next, cells were treated with α-MEM and 10% FBS with or without RANKL (50 ng/mL) and BAG, and the cells migrated for 1 day and 5 days; then, the amount of migration of yellow marked cells in the indicated redline was measured, and Incucyte^®^ Live-Cell analysis systems were normalized percentage based on the only RANKL treated group.

### 4.10. Alizarin Red S Staining

Matrix mineralization in MC3T3-E1 cells was assessed using Alizarin Red S staining purchased from Sigma-Aldrich (Saint Louis, MO, USA). Briefly, MC3T3-E1 cells were washed twice with PBS and fixed with 70% EtOH for 20 min at room temperature. After washing two times with PBS, cells were stained with 40 μM Alizarin Red S at room temperature for 30 min. Staining was visualized using a microscope. Ten random fields were chosen to quantify matrix mineralization using Image J software.

### 4.11. Real-Time Quantitative PCR

Cell lysate was then placed in TRIZOL reagent to extract the total RNA. The concentration of mRNA was analyzed by Nano Drop (Thermo scientific, Waltham, MA, USA). cDNA was synthesized using TOPscript™ RT DryMIX (dT18 plus). Real-time PCR reactions were operated in a LightCycler 480 (Roche, Basel, Switzerland) instrument using TB Green^®^ Premix Ex Taq™ II (Tli RNaseH Plus). The expression levels of Acid Phosphatase 5, Tartrate Resistant (ACP5), ATPase H+ Transporting V_0_ Subunit D2 (ATP6V0d2), dendritic cell-specific transmembrane protein (DC-STAMP) osteopontin (OPN), alkaline phosphatase (ALP), runt-related transcription factor 2 (RUNX2), Osteoprotegerin (OPG) and receptor activator of nuclear factor kappa-B ligand (RANKL) were detected by RT-PCR. Glyceraldehyde-3-phosphate dehydrogenase (GAPDH) was used as the housekeeping gene, and then the mRNA level of each gene was normalized relative to GAPDH. The change in gene expression was calculated using the following equation: 2−ΔΔCT, where ΔΔCT = (CT*target*−CT*gapdh*) at time x−(CT*target*−CT*gapdh*) at time 0, time x represents any time point, and time 0 represents the 1 X expression of the gene in the untreated cells normalized to *gapdh*. All gene expressions were conducted separately 3 times. The primers are presented in Table 2.

### 4.12. Western Blot Analysis

After incubation of SH-SY5y cells in the desired condition, cells were lysed with a lysis buffer (1% Triton X-100, 150 mM NaCl, 20 mM Tris pH 7.5, one tablet of protease inhibitor and one tablet of phosphatase inhibitor) for 30 min on ice and centrifuged at 14,000 rpm for 20 min at 4 °C. Approximately 40 μg of the lysate was loaded on 8–12% SDS–PAGE gels. After running, separated proteins were transferred onto PVDF membrane (Bio-Rad, Hercules, CA, USA). The membrane was blocked in 5% skim milk for 1 h, rinsed and incubated with the indicated primary antibodies in 3% skim milk overnight. Excess primary antibodies were washed out in TBS with 0.1% of Tween 20 (TBS-T) four times and then incubated with 0.1 μg/mL peroxidase-labeled secondary antibodies (against rabbit or mouse) for 2 h. After three washes with TBS-T, bands were visualized with ECL Western blotting detection reagents (Thermo fisher scientific, Waltham, MA, USA) with ImageQuant LAS 4000 (GE Healthcare, Chicago, IL, USA). All observed bands were quantified with ImageJ software and normalized with control.

### 4.13. Translocation Analysis by Western Blot

For translocation analysis, NE-PER nuclear and cytoplasmic extraction reagent (Thermo fisher science, Waltham, MA, USA) was used according to the manufacturer’s instructions. Briefly, cells were lysed in CER buffer for 10 min on ice and centrifuged at 14,000 rpm for 5 min to separate the cytoplasmic extract from the total lysate. After centrifugation, the supernatant was clearly removed and the pellet was dissolved in NER buffer for 40 min on ice. After centrifugation, the supernatant was collected as a nuclear extract. Then, Western blot was performed from above to detect the amount of protein in the cytoplasmic and nuclear extracts.

### 4.14. Statistics

The data were means ± standard deviation (S.D.) of Sigma Plot software 12.1 Student’s *t*-test were used to compare two independent samples. *p* < 0.05 was considered statistically significant.

## Figures and Tables

**Table 1:** ^1^H (700 MHz) and ^13^C (175 MHz) NMR data of BAG in CD_3_OD.

Position	BAG	
	^1^H (*δ*)	^13^C (*δ*)
2	-	157.8
3	-	134.2
4	-	178.0
5	-	161.7
6	6.18 (1H, d, *J* = 2.2)	98.5
7	-	164.4
8	6.31 (1H, d, *J* = 2.2)	93.3
9	-	157.3
10	-	104.2
1′	5.16 (1H, d, *J* = 7.7)	121.8
2′	7.82 (1H, d, *J* = 2.2)	116.2
3′	-	144.8
4′	-	148.1
5′	6.85 (1H, d, *J* = 8.4)	114.6
6′	7.57 (1H, dd, *J* = 8.4, 2.2)	121.8
1″	-	102.9
2″	3.67 (1H, dd, *J* = 9.5, 7.7)	74.4
3″	3.50 (1H, dd, *J* = 9.5, 4.4)	76.8
4″	3.86 (1H, dd, *J* = 4.4, 3.7)	69.9
5″	3.46 (1H, m)	77.1
6″	a: 3.59 (1H, d, *J* = 5.5), b: 3.67 (2H, dd, *J* = 5.5)	61.2

Position (′, ″): The chemical shifts were derived directly from correlation data for position ^13^C d (ppm) ^1^H d (ppm) integration multiplicity coupling J (Hz).

**Table 2:** Primer sequences of Real-time quantitative PCR analysis.

Target Gene	Sequence (5′-3′)	
*ccl4*	Forward	CTCAGCCCTGATGCTTCTCAC
	Reverse	AGAGGGGCAGGAAATCTGAAC
*sod*	Forward	AACCAGTTGTGTTGTCAGGAC
	Reverse	CCACCATGTTTCTTAGAGTGAGG
*cat*	Forward	AGCGACCAGATGAAGCAGTG
	Reverse	TCCGCTCTCTGTCAAAGTGTG
*dcstamp*	Forward	TTTGCCGCTGTGGACTATCTGC
	Reverse	GCAGAATCATGGACGACTCCTTG
*acp5*	Forward	CGTCTCTGCACAGATTGCAT
	Reverse	GAGTTGCCACACAGCATCAC
*Atp6v0d2*	Forward	TGTGTCCCATTCTTGAGTTTGAGG
	Reverse	AGG GTCTCCCTGTCTTCTTTGCTT
*rankl*	Forward	GCTCCGAGCTGGTGAAGAAA
	Reverse	CCCCAAAGTACGTCGCATCT
*runx2*	Forward	CCTGAACTCTGCACCAAGTCCT
	Reverse	TCATCTGGCTCAGATAGGAGGG
*alp*	Forward	ATGGAGGATTCCAGATACAGG
	Reverse	CCATGGTAGATTACGCTCACA
*opn*	Forward	GCTATCACCTCGGCCGTTGGGG
	Reverse	CATTGCCTCCTCCCTCCCGGTG
*opg*	Forward	TCCTGGCACCTACCTAAAACAGCA
	Reverse	ACACTGGGCTGCAATACACA
*gapdh*	Forward	ACAGTCCATGCCATCACTGCC
	Reverse	GCCTGCTTCACCACCTTCTTG

## Abbreviations

ACP5	Acid Phosphatase 5, Tartrate Resistant;
BAG	(2R)-4-(4-hydroxyphenyl)-2-butanol 2-O-β-d-apio-furanosyl-(1→6)-β-d-glucopyranoside
ATP6v0d2	ATPase, H+ transporting, lysosomal V_0_ subunit D2
DC-STAMP	Dendritic cell specific transmembrane protein
OPG	Osteoprotegerin
OPN	Osteopontin
RANK	Receptor activator of nuclear factor kappa-B
RANKL	Receptor activator of nuclear factor kappa-B ligand
RUNX2	Runt-related transcription factor 2
